# Contribution of natural killer cells in innate immunity against colorectal cancer

**DOI:** 10.3389/fonc.2022.1077053

**Published:** 2023-01-04

**Authors:** Zeinab Ghazvinian, Shahrokh Abdolahi, Samaneh Tokhanbigli, Shadi Tarzemani, Andrea Piccin, Mohammad Reza Zali, Javad Verdi, Kaveh Baghaei

**Affiliations:** ^1^ Department of Applied Cell Sciences, School of Advanced Technologies in Medicine, Tehran University of Medical Sciences, Tehran, Iran; ^2^ Basic and Molecular Epidemiology of Gastrointestinal Disorders Research Center, Research Institute for Gastroenterology and Liver Diseases, Shahid Beheshti University of Medical Sciences, Tehran, Iran; ^3^ Northern Ireland Blood Transfusion Service, Belfast, United Kingdom; ^4^ Department of Internal Medicine V, Medical University of Innsbruck, Innsbruck, Austria; ^5^ Department of Industrial Engineering, University of Trento, Trento, Italy

**Keywords:** natural killer cell, colorectal cancer, CAR-NK cell, check point inhibitor, cancer vaccine, cancer immune-cell therapy, adoptive cell immunotherapy

## Abstract

Natural killer cells are members of the innate immune system and promote cytotoxic activity against tumor or infected cells independently from MHC recognition. NK cells are modulated by the expression of activator/inhibitory receptors. The ratio of this activator/inhibitory receptors is responsible for the cytotoxic activity of NK cells toward the target cells. Owing to the potent anti-tumor properties of NK cells, they are considered as interesting approach in tumor treatment. Colorectal cancer (CRC) is the second most common cause of death in the world and the incidence is about 2 million new cases per year. Metastatic CRC is accompanied by a poor prognosis with less than three years of overall survival. Chemotherapy and surgery are the most adopted treatments. Besides, targeted therapy and immune checkpoint blockade are novel approach to CRC treatment. In these patients, circulating NK cells are a prognostic marker. The main target of CRC immune cell therapy is to improve the tumor cell’s recognition and elimination by immune cells. Adaptive NK cell therapy is the milestone to achieve the purpose. Allogeneic NK cell therapy has been widely investigated within clinical trials. In this review, we focus on the NK related approaches including CAR NK cells, cell-based vaccines, monoclonal antibodies and immunomodulatory drugs against CRC tumoral cells.

## Introduction

Natural killer cells (NK cells) as granular cells, consist of nearly 5-15% of peripheral blood lymphocytes. These cells are classified as innate immunity cells since they can create a defensive barrier without previous exposure to a pathogen, cancer cells, or recognition by Major Histocompatibility Complex (MHC) (1).

Human NK cells are divided into two subgroups based on the expression of CD56: CD56 ^bright^ and CD56^dim^ cells. These are functionally and phenotypically different. CD56^bright^ NK cells are mostly found in secondary lymphoid tissues, while cytotoxic CD56^dim^ NK cells are found in circulation (2). Although NK cells belong to the innate immune system, they also have some adaptive immune features.

Previous studies of conducted on CMV infection and on response to activatory cytokines identified two distinct populations of memory NK cells as antigen-dependent and –independent (3, 4).. The strategy to induce memory-like NK cell differentiation is a novel approach for cancer immuno therapy (5). The anti-tumor efficacy of NK cells is dependent to ratio of activating/inhibiting receptors present on their surface. Activating receptors of NK cells are NKG2D, DNM-1, Natural Cytotoxicity Receptors (NCRs) and type 2 receptor (KIR) family. NK activation releases of inflammatory cytokines as well as granules with lytic properties which cause the lysis of tumor cells (6).

One of the problem of immune cell therapy is the fact that NK cells within tumor micro environment (TME) are scanty and always suppressed (7). Tumor cells deceive NK cells in several ways: *a)* by increasing human leukocyte antigen E (HLA-E) (8) and HLA-G (9); *b) via* inhibitory immune checkpoints (10); *c)* decreasing cytokine expression (11); *d)* decreasing NK cell apoptotic activity (12); *e)* reducing expression of activating receptors on NK cells (13); *f)* increasing the expression of prostaglandin E2 by tumor-associated fibroblasts (14).

The main target of CRC immune cell therapy is to improve the tumor cell’s recognition and elimination by immune cells. Adaptive NK cell therapy is the milestone to achieve the purpose. We will review the genetically manipulated NK cells and novel immunotherapy approaches including immunomodulatory drugs, monoclonal antibodies and cancer vaccines that may enhance cytotoxicity of NK cells towards CRC.

## Regulation of NK activity

### Contribution of Receptor- Ligand

**Figure 1:**
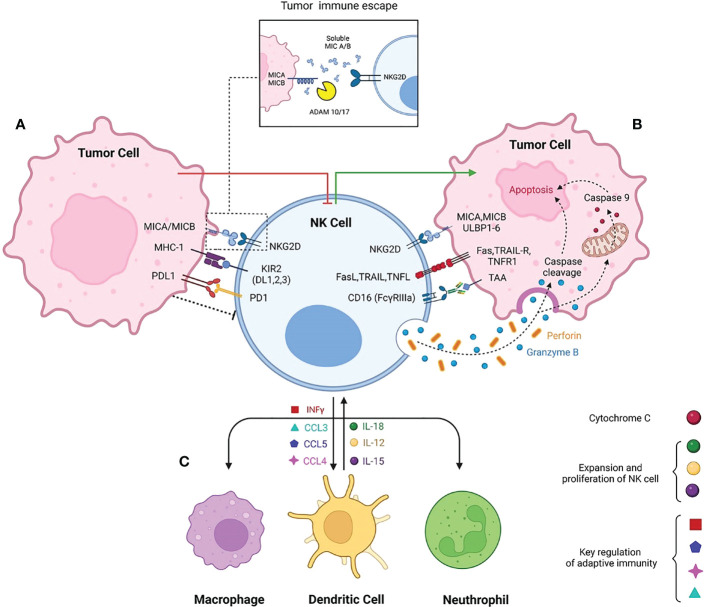
*schematic diagram of Natural killer cell contribution to innate immunity.* The balance of NK cells activatory and inhibitory receptors promotes either secreting cytotoxic molecules or making NK cells exhausted in TME respectively. **(A)** Immune suppressive TME is due to interaction of MHC-1 with inhibitory receptors such as KIR2. High expression of PDL-1 in tumor cells interacts with inhibitory checkpoint PD-1, inhibiting the NK cell activation. Beside the activatory role of MICA/MICB, the shedding form caused by ADAM17/10 cleavage makes the NK cells non responsive to tumor target cells. **(B)** NKG2D the most prominent activatory receptor of NK cells interact with MICA/MICB/ULBP. Following NK cells activation Fas ligand, TRAIL and TNF ligand highly express on NK cells to induce the apoptosis independent of perforin and granzyme. Antibody dependent cytotoxicity of NK cells promotes perforin and granzyme activation and consequently granzymes triggers apoptosis even dependent or independent of caspase cleavage. **(C)** Interaction of NK with other immune cells. NK cells secret INFγ, CCL3, CCL5, CCL4 to contribute with macrophages, dendritic cells and neutrophils. The innate immune cells subsequently secret IL-15, IL-18 and IL-12 to provoke NK cells expansion and activation.

Activator and inhibitory receptors on NK cells detect the protein ligands on the infected and tumor cells. The ratio of these receptors will determine the activation or inhibition of NK cell cytotoxic cascades. Most healthy human nucleated cells present MHC class I loaded with self-peptides that are inhibitory ligands and will switch off NK cells. Thus, the suppression/expression of MHC molecules on the surface of most tumor cells play a key role in NK activation (**Figure 1**). KIRs are the key MHC receptors and regulate the development, activation, and cytotoxic features of NK cells. There are various isoforms of KIRs that, in different physiologic and pathologic conditions may play the role of inhibitory or activator receptors. For example, it is reported that the KIR2DL1 isoform is specific against HLA-C2, consisting of an Ig-like inhibitory motif (15). However, KIR 2DS5, 2DS1, and 3DS1 containing Ig-like activatory motifs are associated with an increased complete remission post-chemotherapy in metastatic CRC (16). HLA-E is a non-classical member of the MHC class-I family that presents self-peptide on almost all nucleated cells and in normal conditions, will dampen NK cell activation by interaction with NKG2A inhibitory receptor. However, in the tumor context of CRC, over-expressed HLA-E is an immune escape mechanism that inhibits NK cell activation through high-affinity interaction with the NKG2A receptor (17, 18). Activator receptors on NK cell surface play an essential role in tumor cell recognition and elimination. NKG2D is a critical activating receptor of NK cells and its expression will be upregulated during NK cell maturation and activation (19). NKG2D ligands are MHC class-I chain-related A and B molecules, also known as MICA/MICB and UL binding protein 1-6 (ULBP1-6). NKG2DLs are not expressed under normal condition on healthy tissues, and their expression is intracellular mainly (20). However, their expression on cell surface has been reported in various carcinomas due to cellular stress (21), including CRC. The same are also reported as a good prognostic biomarker (22, 23). Besides surface expression of NKG2D ligands on tumor cells, there are shedding form of MICA and MICB derived from transformed cells in TME (20). Continuous exposure of NKG2D with soluble forms of MICA and MICB results in reduced NK cells cytotoxicity, downregulating NKG2D expression. Altogether, these induce tumor cells proliferation (24). Soluble forms of NKG2DLs is due to protease cleavage of their conserved motif in the α3 domain. Two dis-integrin and metalloproteinase enzymes known as A Dis-integrin and Metalloproteinase domain-containing protein 10 and 17 (ADAM10 and 17) are crucial in making soluble forms of MICA/B, and ULBP (25). It is reported that platelets are one of the secretory origins of ADAM10 and 17. In metastatic lung cancer, these soluble platelet-derived factors impair the NK immune surveillance towards tumor cells (26).

The other family of no MHC restricted NK receptors are Natural cytotoxicity receptors (NCRs), including NKp46, NKp30, NKp44, and NKp80. NCRs ligands are expressed on viral infected or tumor cells and extracellular soluble forms (27). The density of NCRs on NK cells’ surfaces is coordinates with NK cells activation and cytotoxic function (28).

Receptor-ligand modulation may impair NK cells functionality in tumor elimination. Novel therapeutic strategies would selectively target this approach of NK cells in tumor context. The interactions between NK Cell Receptor and human target cell ligands are summarized in **Table 1**.

**Table 1:** Interaction of NK cells’ receptor with tumor cells’ ligand.

Natural Killer Cell Receptors	Receptor Type	Human Target Cell Ligand	Ref
KIR2DL1, KIR2DL2, KIR2DL3	Inhibitory motif (Ig-like domain)	HLA-C1,HLA-C2	(29)
KIR2DL4	Inhibitory motif (Ig-like domain)	HLA-G	(29) (30)
KIR2DL5A, KIR2DL5B	Inhibitory motif (Ig-like domain)	Unknown	(31)
KIR3DL3	Inhibitory motif (Ig-like domain)	B7 family member HHLA2	(31) (32)
KIR2DS1,KIR2DS2,KIR2DS3, KIR2DS4, KIR2DS5	Activatory motif (Ig-like domain)	HLA-C	(31)
KIR3DS1	Activatory motif (Ig-like domain)	HLA-B Bw4-I80	(33) (34)
NKP30	Activatory motif (Ig-like domain)	Soluble BAG6,B7H6,Viral HA	(35)
NKP44	Activatory motif (Ig-like domain)	PCNA, Viral HA	(35)
NKP80	Activatory motif (C-type Lectin Domain)	AICL	(35)
NKp46	Activatory motif (Ig-like domain)	Viral HA,vimentin,heparin sulfate	(36–38)
CD94-NKG2C/E/H	Activatory motif (C-type Lectin Domain)	HLA-E	(39)
CD94-NKG2A	Inhibitory motif (C-type Lectin Domain)	HLA-E	(39)
NKG2D	Activatory motif (C-type Lectin Domain)	MICA,MICB,ULBP-1-6	(29)
CRTAM	Activation of NK cell migration (Ig-like domain)	Necl-2/IGSF4A	(40)
DNM-1/CD266	Activatory motif (Ig-like domain)	CD112(Nectin-2),CD155(PVR)	(41)
TIGIT	Inhibitory motif (Ig-like domain)	CD112,CD113(Nectin-3),CD155	(42)
CD96	Inhibitory motif (Ig-like domain)	CD155,CD111(Nectin-1)	(42)
2B4/CD244/SLAM4A	Adaptor molecule dependent (whether activatory or inhibitory) (Ig-like domain) (GPI linkage)	CD48	(42)
NTB-A/SLAMF6	Co-receptor of NK cell activation (Ig-like domain) (GPI linkage)	NTB-A/SLAMF6	(43)
CRACC/SLAMF7	Activatory receptor (Ig-like domain) (GPI linkage)	CRACC/SLAMF7	(43)
FcR gamma	Activatory motif (Ig-like domain)	IgG	(44)
CD27	Activatory receptor (EC cadherin Domain)	CD70/CD27L	(45, 46)
CD100/Semaphorin 4D	Indirectly activates NK cell cytotoxicity (Ig-like domain)	CD72	(47, 48)
CD160	Activates cytolysis effects of NK cells (Ig-like domain)	HLA-C	(29–49)
Tim-3	Inhibitory motif (Ig-like domain)	CEACAM-1,CEACAM-5,galectin9.HMGB1	(50)
ILT2/LILRB1	Inhibitory motif (Ig-like domain)	HLA classI	(51)
KLRG1	Inhibitory motif (C-type Lectin Domain)	E-N-R Cadherins	(51)
LAIR1	Inhibitory motif (Ig-like domain)	Collagen	(51)
CD161/NKR-P1A	Inhibitory motif (C-type Lectin Domain)	OCIL/CLEC2d	(52)
Siglec-3,Siglec-7,Siglec-9	Inhibitory motif (Ig-like domain)	Sialic Acid	(53)

### Cytokines induce NK cells’ cytotoxicity

Besides receptor-ligands interactions, cytokine secretion and exposure are demanded for NK cell activation (54). Several cytokines have been reported to modulate NK cell activation and proliferation. Cytokine effectiveness in NK cells has been reported mainly through JAK/STAT, CIS, and SOCS signaling pathways (55–57). The crucial cytokines activating NK cells are: IL-2, IL-12, IL-15, and IL-18. These molecules trigger their effects thanks to signal transducer and activator of transcription (STAT) proteins (58). The suppressor cytokine signaling 2 (SOCS2) inhibits JAK/STAT signaling and with negative feedback, regulates NK cell differentiation. NK cells’ cytokine signaling modulate homeostasis with IL-2, IL12, IL-15 and IL 18 and limit INFα signaling through STAT1 (59). Immature NK cells develop to mature NK cells through the acquisition of activator receptors such as NKG2D. IL-2/IL-15/IL-18 increases the cytotoxic activity of NK cells by NKG2D over expression (60). IL-12 and IL-2 enhance the NKG2D signal activation. However, IL-2 exclusively effects the activation of CD56 ^bright^ NK cells (61).

Moreover, IL-2 and IL-15 induce NKG2D, KIRDL1, and KIRDL2 expression on NK cells, T cells, and NKT cells of regional lymph nodes. This enhances NK cell cytotoxicity and increases the anti-tumor potential of innate and adaptive immune cells (62).

A cocktail-based strategy by utilization of IL-2, IL-15, and IL-18 cytokines was established to generate *in vitro* peripheral blood-derived NK cells with a strong cytotoxic effect (63). Systemic administration of IL-2 in cancer patients improved survival rate, however high doses was reported to be toxic (64). Intratumoral administration of IL-2 is an alternative method to systemic therapy, but this is limited to accessible tumors (65). Targeted delivery of IL-2 is a novel approach to enhance the cytotoxicity effects of tumor resident NK cells. Highly preserved glycoproteins such as fibronectin expressed in solid tumors neo-endothelial could be a target of cancer therapy. L19-IL-2 is an immune-cytokine that targets extra domain of fibronectin fused with IL-2. The IL-2 accumulates selectively in the tumor site, boosting the tumor immune response (66).

IL-15 is an immuno-stimulatory cytokine that promotes the survival and proliferation of NK cells. IL-15 receptor is a heterotrimeric complex consisting of IL-15R α, IL-15R β and common γ chain (67). Antigen-presenting cells secret IL-15/IL15Rα in two forms soluble and membrane-bound. IL-15/IL-15Rα subsequently transduces signaling through four independent mechanisms known as IL-15 trans presentation delivery (68, 69). Recombinant IL-15 had been reported to evoke anti-tumor activity of adaptive and innate immune cells in colon cancer (70) however, Short half-life and poor bioavailability limit the therapeutic potential of IL-15 systemic therapy.

It is reported that chimeric IL-15 (covalently linked IL-15 with IgG2) improves IL-15 half-life, enabling greater bioavailability for an extended period of time (71). As the delivery of IL-15 is reported through trans presentation, a novel approach investigated an immune cytokine known as KD033 that both targets PDL-1 and directly delivers IL-15/IL-15Rα complex to immune cells resident in TME. 7 days after a single dose of KD033, a dose-dependent increase in peripheral NK cells and NKT-like cells were induced (72). Furthermore, Rui Ma; et al. developed a new oncolytic herpes virus expressing IL-15/IL-15Rα and demonstrated in a murine glioblastoma model therapeutic efficacy (73).

Although there is the beneficial roles of IL-15 in immune cell stimulating, the secretion is regulated through the cytokine induced SH2-containing protein (Cish) that provides negative feedback by preventing the JAK/STAT5 signaling (57). Following CIS deletion in NK cells, hyperactive IL-15 signaling leads to increased activation of the AKT/mTOR and c-Myc signaling, inducing a high proliferation rate of NK cells (74). IL-21 and 18 are stimulate NK cell proliferation and activation. The signaling through a typical γ chain subsequently induces INFγ production in mature NK cells (75). Targeted cytokine delivery, in contrast to systemic therapy, will improve the beneficial effects of treatment with minimum side effects. A fusion of anti-PD-1 antibody and IL-21 inhibits the PD-1 immune checkpoint signaling and delivers IL-21 to targeted receptors, improving treatment efficacy (76). IL-18 receptor is highly expressed in NK cells. However, most tumors express IL-18 decoy receptor in TME, limiting the anti-tumor immunity of therapeutic recombinant IL-18. Ting Zhou; et al. have engineered a decoy resistance IL-18 receptor (DR-18); that maintains the IL-18 signaling and the compound is resistant to IL-18-decoy receptor inhibition protein. These results demonstrated that DR-18 enhanced the maturation and activation of NK cells in anti-PD-1 resistance tumors (77).

Besides the investigation of cytokines effects one by one, the compound of cytokines profile plays an essential role in the generation of different NK cell subtypes. Cooper et al. first studied cytokine-induced memory-like NK cells in 2009. They reported that IL-15/IL-18/IL-12 pre-activated NK cells showed higher INFγ production by cytokine profile re-stimulation, in contrast to pre-activated group (4). The memory-like NK cells are phenotypically different from classical cytotoxic NK cells (78). Low dose of IL-2 activates the cytokine-induced memory-like NK cells more prominently (79). Significantly metabolic features of cytokine-induced memory-like NK cells will be reprogramed during pre-activation. mTOR controls metabolic reprogramming and cytotoxicity function of NK cells. This gene will be activated during cytokine exposure (80). Due to their high cytotoxic activity and anti-tumor properties, cytokine-induced NK cells represent the novel immune cell therapy against tumors. However, their therapeutic potential may be further improved studying how they change after cytokine exposure, combination immunotherapy approaches, genetically modifications (81). Generally, the anti-tumor activity of NK cells is based on the responsiveness to growth factors and cytokines that can extremely influence the tumor immune surveillance. The specific contribution of NK cells and cytokines still needs to be better identified.

### Cytoskeleton as a regulator of NK cells’ immune responses

NK cells degranulation is a multi-step process. This consists of: *a)* immunologic synapse formation by ligand-receptor molecules of NK cells and tumor cells; *b)* polarization of lytic granules toward the immunologic synapse, and directly release of cytotoxic granules toward target cells (82).

Cytolytic granules with acidic pH are kind of lysosomes that contains pore-forming molecule, granzyme, Fas Ligand, and granulysin in NK cells. Perforin is a pore-forming molecule, while granzymes are a serine protease family that induces programmed cell death (apoptosis) in tumor cells (83). Perforin mediates target cell membrane pore forming in a pH and calcium dependent manner, consequently delivering the serine protease granzyme toward the target cell (84).

NK cells are one of primary cells that induce the extrinsic apoptosis pathway by expressing tumor necrosis factor super family members such as Fas ligand (FasL), and TRAIL. Interaction of death receptor ligands will recruit adaptor proteins such as FADD (Fas-associated protein with death domain), TRADD (Tumor necrosis factor receptor type 1-associated death domain protein), and a series of downstream factors, including caspase-3, -6, -7, -8, -9 eventually leading to apoptosis (85). Independent caspase apoptosis mediated by granzymes is reported by provoking mitochondria’s permeabilization by with cytochrome C release in the cytosol and apoptosome complex formation (86).

The cytoskeleton can regulate NK cells’ immune response. NK cell migration and immune synapse formation are due to polymerization and depolymerization of actin proteins. F-actin filaments will polymerize in response to receptor-ligand interaction and trigger the downstream signaling pathways, including PLCγ, MEK, and ERK (87). Besides NK cells receptor-ligand interactions, monoclonal antibodies have been reported to induce NK lytic granule polarization by activating PLCγ, MEK, and ERK signaling pathways (88). Cytotoxic granule release is highly dependent from F-actin generation due to the mediator of actin regulatory protein, EVL (89). Recently, filamin A (FLNa) protein cross links F-actin filaments was reported playing an essential role in the degranulation of NK cells following synaptic accumulation of F-actins (90). In addition to actin proteins, myosin IIA is the next cytoskeleton protein that promotes NK cells functionality by utilizing ATP to induce the contractile force on F-actin filaments (91). Moreover, microtubule filaments consist of alpha and beta tubulin heterodimers, and utilize microtubule-associated proteins (MAPS) to facilitate the delivery of NK cells’ lytic granules in the immunologic synapse (92).

Cytoskeleton machinery abnormalities impair NK cell migration and cytotoxic activity. The relationships between NK cells and cytoskeleton should therefore be deeper investigated (93).

## Colorectal cancer and impaired NK cell function

The mechanisms sustaining inflammation in CRC are not fully understood. In colitis-related colon cancers, chronic inflammation plays an influential role in the progression of the disease to malignancy (94). Innate immune system cells, including neutrophils, macrophages, NK cells, and B- and T-cells of acquired immunity, are involved in post-inflammatory cancer processes (95). According to studies conducted on CRCs, inflammation and the presence of NK cells at the tumor site play an essential role for the progression of the disease (96). In CRC microenvironment NK cells and TCD8^+^ cells interact through secretion of INFγ, IL-2, and HMGB1 (high-mobility group protein 1). Cross-talk between NK, TCD8+, and M1 cells are involved in the induction of a pro-inflammatory immune response that leads to the activity of TCD8^+^ cells in CRC microenvironment. IL-2 activated NK cells, secret INFγ, and Leptin. Leptin directly stimulates M1 cells to produce IL-1β. Subsequently, T cells synthesize the pro-inflammatory cytokine IL-6. INFγ indirectly induces IL-6 production from the macrophage (97).

Activated NK cells produce CXCL1, CCL1-3-4-5, CCL-22, and CXCL-8, chemo-attractants for other immune cells, and recruit them to the tumor site. A preclinical study in a colorectal mouse model reported that the secretions of CCL-3 from CT26 tumor cells change the inflammatory response in the early phase of tumor response. It causes mobilization of B, T-cells, dendritic cells, and CD49+ NK cells to the tumor site (98). There are differences in tumor-infiltrating immune cells among CRC on the left and right sides. Left-side tumors (LCC) are associated with a high number of CD56 ^bright^ NK cells that correlates with patient survival (99). Left-side tumors show a higher rate of response to therapy (100). It is demonstrated that CD56^dim^ LCC infiltrated NK cells could be a prognostic biomarker in CRC (101). Mc Gilvray et al. showed NKG2D ligand expression involves in cancer immunosurveillance and associated with Prognosis (102). Furthermore, NK cells express programmed cell death protein 1 (PD-1) substantially, and is further increased after stimulation and indicates poor prognosis in digestive cancer patients (103).

Various studies have been conducted on the infiltration rate of NK cells which showed that NK cells infiltrating solid tumors were relatively low and demonstrated that the NK cell number was too low to pursue prognosis (104–107). Moreover, Sarah Nersesian et al. investigated the prognostic value of NK cells for solid tumors. They reported 1.9% found a negative association between overall survival and NK cell infiltration and 38.9% reported no impact and 59.3% positive associations between NK cell infiltration and overall survival (108). These reports demonstrate NK cells as a positive prognostic factor in solid tumors.

There are 2 types of activated macrophages: *a)* activated M1, which participates in immune response, and *b)* activated M2, which promotes tumor progression. Tumors-associated macrophages (TAMs), more similar to the M2 subtype, are the major players of cancer-related inflammation (109) and play a crucial role in NK cell suppression in the tumor milieu of CRC. Cancer-associated fibroblasts (CAFs) by enhancement of TAMs impair the NK cell function (98).

Elevated TGF-β in patients suffering metastatic CRC is reported (110). TGF-β receptor mutation causes CRC with microsatellite instability (MSI-high). This kind of mutation, is associated with better survival (111). TGF-β has been showed to impair NK cell cytotoxicity in a CRC mouse model. LY2157299, a TGF-β receptor kinase inhibitor, combined with adoptive NK cells, eradicated the liver metastasis of colon cancer in a mouse liver metastasis CRC model (112).

**Figure 2:**
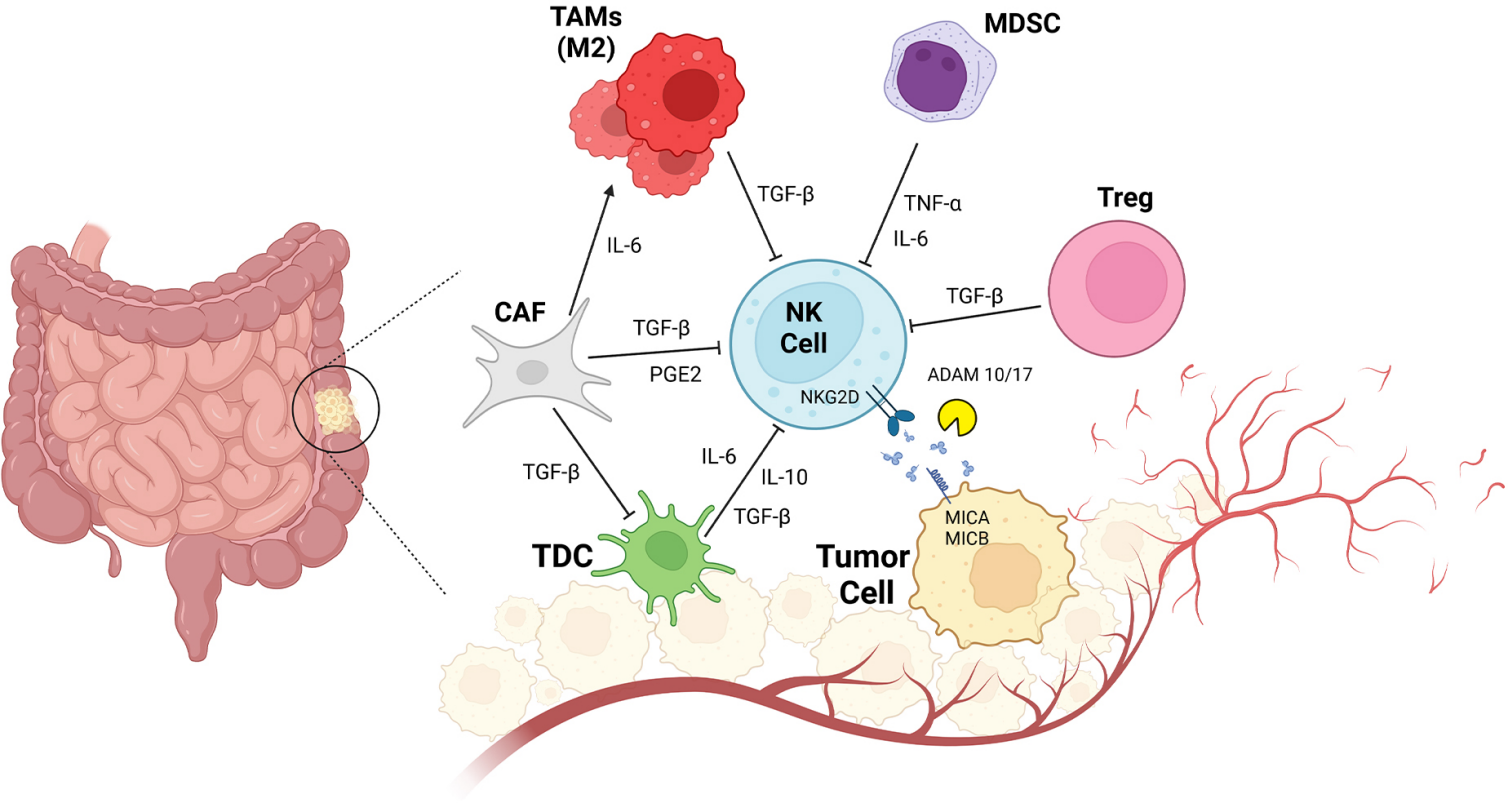
*CRC and Impaired NK Cell Function due to innate immune cell interactions.* Tumor resident innate immunity cells consist of CAF (cancer associated fibroblast), TAMs (tumor associated macrophage), MDSC (myeloid derived suppressor cells), Treg (regulatory T cells), tDC1(conventional Type1 dendritic cells) interact with NK cells. TGF-β secreted by CAf, TAMs, Treg inactivates NK cells and also DCs in TME. Other regulatory cytokines such as IL-6, IL-10 and TNFα also inhibits NK cells activation. High PGE2 levels makes the NK cells non-responder to tumor cells. Shedding forms of MICA/MICB is a tumor cell direct mechanism to escape from NK cells immune response.

NK cell manipulation would be a promising target in the CRC immunotherapy context (113). As mentioned here, NK cells are innate immune cells that, without previous exposure to tumor antigens and HLA-priming, provide immunity against tumor cells (114). However, in CRC, NK cell dysfunction represents an immunological failure. NK cell dysfunction allows escape of tumor cells in colorectal, gastric, and pancreatic cancers. In all types of malignancies, a marked decrease in NKP30 NK cells has been reported (115). In cancer, several mechanisms impair NK cell function, for example: *a)* decreased NK cell count and *b)*modified phenotype, and impaired function due to inhibitory interactions with other immune cells presented in TME (**Figure 2**) (116).

NK cell count is an independent prognostic factor in CRC (11). Immune cell profile in peripheral blood of CRC patients can be a prognostic factor. It was shown an increasing percentage of circulating Tregs and a reduction of CD56^dim^ NK cells in CRC patients. The low rate of CD16^+^ NK cells has been associated with shorter disease-free survival (DFS) (117). In chronic inflammation, mediators such as Prostaglandin E2 (PGE2) are associated with worse survival of CRC patients. PGE2 and upstream enzymes called COX (Cyclooxygenase) linked to CRC were first studied in 1994 (118). Acid arachidonic metabolizes to PGE2 through the COX pathway. PGE2 mechanism in CRC had been reported to stimulate cell invasion, tumor growth, and apoptosis inhibition (119). When PGE2 is produced in high amounts has an immunosuppressive role in TME produced by either immune cells and tumor cells (120). PGE2 has adverse effects on NK cell function, survival, and proliferation in tumor sites (121). PGE2 downregulates NK cell-activating receptors through cAMP-mediated PKA type I-dependent signaling (122), and it regulates IL-12 and IL-18 dependent INFγ synthesis in NK cells (123). A COX-competent animal model of CRC showed increased secretion of prostaglandin E2 and a lack of conventional type 1 dendritic cells (cDC1) mobilization to the tumor site. PGE2 impaired NK cell accumulation and its ability in tumor site to produce CCL5 chemokine. PGE2 also reduces the expression of CCL5 and CXCL1 receptors on cDC1 cells (124). Inhibiting COX2 activity by andrographolide (a natural diterpenoid from *Andrographis Paniculata*) decreased PGE2 release. However, the same, improved PD-1 immunotherapy in the xenograft model of CRC by enhancing the functionality of TCD4^+^ and TCD8^+^, enhancing INFγ, and increasing cytotoxic molecules such as perforin and granzymes (125). A recent case control study showed PGE2 plasma level is associated by CRC risk so the nonsteroidal anti-inflammatory drugs (NSAIDs) would be a promising personalized medicine in CRC treatment (126).

TME in CRC is a hallmark of cancer progression, immune cell dysfunction, and immunotherapy resistance. There are various physical and chemical alterations in cancers’ microenvironments, such as hypoxia, acidosis, increased extracellular matrix rigidity, and high interstitial fluid pressure (127). The acidic TME is the critical barrier created by tumor cells against immune cells. Acidification of the tumor site is caused by rapid cancer cell proliferation, a high glucose glycolytic metabolism ratio, and increased lactic acid production (128). Metastasis, tumor progression, immune cell suppression, and poor prognosis are sustained by acidotic processes (129). Acidosis in CRC contributes to tumor progression and resistance to conventional treatments (130). It is shown that lactate accumulation in metastatic CRC induces mitochondrial dysfunction and apoptosis in NK cells; it seems metabolism targeting in CRC is a promising therapeutic approach to overcome immune suppression (131). Targeting acidosis to overcome the resistance to anti-PD-1 and anti-CTLA-4 could be promising (132).

Taken together, NK cells dysfunction from different aspects play an important role in CRC immune response. NK cells exert their cytotoxic function toward impaired cells; and through cytokines and chemokines modulate the adaptive immunity too. However, NK cells fail to infiltrate properly in tumor microenvironment, and when reach the tumor site, they would be exhausted due to immune checkpoints inappropriate expression. NK cells dysfunction in tumor immunity provides the basis of new strategies to harness their immune response.

## Immunomodulatory drugs potentiate NK cytotoxicity against CRC

Immune cell therapy has become a milestone in cancer therapy; however, the immunosuppressive status of the TME is one of the main barriers for the immune system’s function (133). Therefore, interfering with the TME seems to be the clue to restore the immunologic responses (134). Immunomodulatory drugs may interfere with the immunosuppressive status of the TME.

Immunomodulatory drugs that induce CD 8^+^ T cells proliferation can enhance cytotoxicity, activation of DCs, Th1 responses, augmentation of NK cells activity, inhibiting tumor angiogenesis, and changing the production of DCs cytokine profile (135, 136). The cross-talk between these drugs and the NK cells is mediated by IL-2 and IFNγ (137). Thalidomide, lenalidomide, and pomalidomide are examples of these drugs with anti-cancer functions. The mechanism of action of these drugs is anti-proliferative, anti-angiogenic, and proapoptotic (131, 132). The effect of these drugs has been intensively studied in acute myeloid leukemia (AML) as monotherapy or in combination with other therapies. In mice animal models where CRC was induced with CT26, lung metastasis was significantly reduced after treatment with Thalidomide, lenalidomide, and pomalidomide. Interestingly, the pre-treatment of the CT26 cell line with lenalidomide reduced the lung metastasis indicating the anti-metastatic effect of this drug (138).

These immunomodulatory drugs have also opened their way to clinical trials. For instance, in a phase II clinical study, lenalidomide was used in combination with Cetuximab in KRAS mutant patients. Lenalidomide significantly increased the number circulating NK cells. The combination of Cetuximab and Lenalidomide increased granzyme-positive NK cells more than lenalidomide only (139).

Thalidomide monotherapy or chemotherapy showed a mild effect on patients with advanced CRC. Thalidomide may affect NK cells’ cytotoxicity by increasing IL-2 and INFγ production (140, 141). Pomalidomide in CRC preclinical setting and *in vitro* effectively restrained the cytotoxicity of NK cells (142). However, no clinical trial with a pomalidomide-based regime has been conducted in CRC patients so far.

Immuno modulatory drugs reported positively effect on NK cells cytotoxicity by alter cytokine production, NK cells augmentation and decrease metastasis in CRC patients.

## Immune checkpoint blockades improved NK cell cytotoxicity in colorectal cancer

NK cells clearly exerting cytotoxic activity against cancer cell and are a key-player of immunotherapy (143). Stimulatory and inhibitory receptors regulate the cytolytic functions of NK cells. Many of these receptors are expressed by NK cells to mount effective anti-tumor immunity (6). The activation receptors present on the NK cell surface, NCRs, include various receptors such as NKp46, NKp30, NKp44, NKG2D, and DNAM-1 (144). Tumor cells and TME adopt different approaches to evade NK cells’ immune responses and surveillance. KIRs, LIRs, NKG2A, and the classical CTLA-4 and PD-1 receptors to recently discovered B7-H7 are immune checkpoint inhibitors that compromise NK cell anti-tumor activity (145). Anti-PD1, Dostarlimab, has been reported to induce complete response with no evidence of tumor in mismatch repair-deficient, locally advanced rectal cancer patients. A clinical study (NCT04165772) was initiated in 2019, and the results demonstrated the complete treatment at least in a 6-month follow-up (146).

Therefore, targeting these inhibitory immune checkpoints, is proposed as one of the immune therapy strategies to augment NK cell effects (147).

Ipilimumab, a CTLA-4 inhibitor monoclonal antibody, is the first immune checkpoint inhibitor to gain approval with outstanding results in different cancers (148). It has been demonstrated that blocking CTLA-4 has increased the expression of IL-2 and enhanced the cytotoxicity of NK cells (149). Ipilimumab has entered clinical trials combined with nivolumab, the anti-PD-1, to treat metastatic CRC (150). Other immune checkpoint inhibitors in metastatic patients are the NKG2A inhibitor, Monalizumab, and PD-L1 inhibitors, Durvalumab, with no severe side effects, mortality, and practical clinical outcome (151). TIGIT is another cell surface inhibitor on NK cells and T cells, causing exhaustion of TIL and tumor-infiltrating NK cells. Either NK deficient TIGIT or using Blockade of this inhibitor alone or combined with Blockade of PD-L1 significantly restored the suppressed anti-tumor immunity by NK cells in CRC animal models and patients (10).

Blockade of immune checkpoints regulates the cytotoxic activity of NK cells and blockade drugs have been approved in so many malignancies; however, some patients are non-responder to these novel treatments that fundamental studies should be further investigate the effective therapies. A comprehensive overview of the immune checkpoint inhibitors and their Blockade is presented in **Table 2**.

**Table 2:** Immune checkpoint inhibitors present on NK cells and their blockade under clinical and preclinical investigations in CRC.

Immune checkpoint inhibitors	Immune checkpoint inhibitor blockade	Disease classification	Ref
KIR (CD158)	PH2101 Lacutamab Lirilumab	Advanced/Metastatic solid tumors	(152)
PD-1	Nivolumab Durvalumab Dostarlimab Tislelizumab Spartalizumab Sym021 Pembrolizumab Toripalimab Camrelizumab Dostarlimab	dMMR/MSI-H mCRC Locally advanced rectal cancer	(150, 153–155) NCT03927898 NCT04202978 NCT04165772
CTLA-4	Ipilimumab Tremelimumab	dMMR/MSI-H mCRC/refractory CRC	(150, 156)
NKG2A	Monalizumab	recurrent or metastatic CRC	NCT02671435
LAG-3	Relatlimab Eftilagimod (IMP321) MK4280 LAG525	MSS colorectal adenocarcinomas	NCT03642067 NCT05328908 NCT05064059 (157)
TIM-3	Cobolimab LY3321367 BGB-A425 MBG453	Advanced metastatic tumors including CRC	(158–160)
TIGIT	Tiragolumab Etigilimab AB154	(MSI) and MSS-CRC	(161, 162)

defective mismatch repair (dMMR), High microsatellite instability (MSI-H), metastatic CRC(mCRC), Microsatellite stable (MSS).

## Antibodies increase NK cell cytotoxicity against colorectal cancer

Antibody-dependent Cellular cytotoxicity (ADCC) is a mechanism of NK cells’ defense against tumor cells independent of MHC recognition and complement system activation. Antibodies even derived from adaptive immunity activation (a classic form of ADCC) or commercial types (monoclonal antibodies) elicit the cytotoxic activity of NK cells. NK cells interact with antibodies through FcγRIIc/CD32c and FcγRIIIa/CD16a receptors that bind to antibodies Fc portion. IgG1 sub-type comprises high affinity to FC receptors and mediates the NK cells’ ADCC activity. CD16a cross-links on NK cells following antibodies binding (163, 164). CD16a and CD32c are associated with the CD3-ζ chain containing ITAMs (Immune tyrosine-based activating motifs) residue in the cytoplasmic tail. Phosphorylated ITAMs result in NK cell degranulation, cytokine release, and tumor cell lysis by inducing TNF, FasL, and TRAIL death receptors, IFNγ secretion (165), granzyme and perforin release, caspase 8 activation and apoptosis (166). Besides induced ADCC by classical form and monoclonal antibodies, genetically manipulated NK cells expressing high-affinity CD16a will also trigger ADCC more prominently. Reportedly engineered NK-92 cell lines derived from human lymphoma expressing high-affinity CD16a, producing high amounts of IL-2 and granzymes, are highly cytotoxic against colon cancer cell lines (167).

Commercial immunoglobulin molecules are promising approaches in CRC treatment since they interfere with tumor angiogenesis and immune system modulation and induce direct immune cell cytotoxicity through NK cells (168).

CRC disease progression is highly dependent from two signaling pathways; EGFR and VEGF (169). The trastuzumab (anti-HER2), cetuximab (anti-EGFR), panitumumab (anti-EGFR), ramucirumab (anti-VEGFR2), and bevacizumab (anti-VEGF-A) are commercial specific monoclonal antibodies that are currently used in CRC treatment (170). High expression of HER-2 in CRC-derived cancer stem cells was reported with activation of the PI3K/AKT pathway exacerbating tumor cell proliferation. Recent findings showed that monoclonal antibodies in combination with other immune therapy increase treatment efficacy significantly in CRC. HER-2 targeting in combination with PI3K and MEK inhibitors induces tumor regression in avatar models of CRC (171). Cetuximab beneficially intensifies the NK cell cytotoxicity against CRC nude mice model (172).

Cetuximab combined with nivolumab was reported to be well tolerated with less efficacy in metastatic colorectal cancer (166). Panitumumab, combined with standard chemotherapy regimens (FOLFOX), is the first-line treatment in RAS mutated colorectal cancer. Re-treatment, even in the second or third line, could have potential benefits (141). The immunoglobulin backbone of panitumumab that has a low binding site affinity for CD16a, does not induce NK cells mediated ADCC (173). VEGF or VEGFR targeting is the other approach to monoclonal antibody treatment in CRC. Ramucirumab is an IgG1 monoclonal antibody targeting VEGFR-2 and is reported to be a prominent treatment in combination with chemotherapy (FOLFIRI regimens) for second and late-line treatment of metastatic CRC by inhibiting the tumor angiogenesis, improving patients overall survival (174, 175). However, it has not been reported that ramucirumab enhance NK cells’ ADCC.

Bispecific antibodies contain two binding sites against two different antigens. Some bispecific antibodies connect immune cells to tumor cells, triggering the immune cells cytotoxicity. Other bispecific antibodies can target two check points in tumor cells. Additionally, some are designed to concurrently target tumor-associated antigens and check points (176–178). Duligotuzumab, a bispecific antibody against EGFR and Her3, contains FC domains activating NK cells’ ADCC (179). One of the BIKE (bispecific NKcells’engager) antibodies recognizing CD16 on NK cells and EPCAM on tumor cells that facilitates ADCC but not the proliferation of NK cells was engineered by Joerg.U and colleagues in 2013 (180). This group incorporated a modified IL-15 cross-linker to the previous BIKE construct to create a TriKE (trispecific construct) which can improve activation, proliferation, and survival of NK cells (181). Patient derived xenograft models effectively simulate the tumors. These modelsare used to investigate the antitumor effects of immune cell therapy in combination with other agents (182). An antibody featuring simultaneous identification of two variants of CD20 and CD16 [(CD20)2xCD16] is one of the major trispecific antibodies that can effectively activate NK cells’ ADCC. More over this can be a mediator of malignant B-cells’ lysis in animal model (183).

Cytokines also play an essential role in activating NK cells’ ADCC; they are important mediators in tumorigenesis and would be used as anti-cancer treatments in CRC (184). Co-administration of IL-21 with cetuximab in phase I clinical trial of CRC increased NK cell-mediated ADCC (178). Cetuximab in combination with IL-2 and IL-15 improved the cytotoxicity of dysregulates blood NK cells in CRC patients (185). Moreover, combination therapy of rituximab and IL-2 demonstrated a synergistic role in activating NK cells (186). ALT-803 and IL-15 super agonists have also been reported to increase NK cell-mediated responses through CD16a, thus inducing ADCC (187, 188).

Following preclinical research, the synergy potential of targeted antibodies with NK cell therapy should be further investigated in clinical trials. Our research group demonstrated that pretreated checkpoint blockade NK cells could effectively enhance the NK cells’ trafficking in TME and beneficially reduce the tumor cell mitosis in gastric cancer xenograft model (189). However the same results was not achieved in chemo immune cell therapy of Intratumoral injection of NK cells in combination with capecitabine in gastric cancer xenograft model in the other study of our group (190).

## Vaccines augment anti-tumor immune responses against colorectal cancer

In cancer cells, many proteins look similar to healthy proteins, which keeps the cancerous cells out of the immune system’s reach. Vaccines expose antigens to the immune system and this will trigger the immune response. Identifying the exact antigens in different cancer types is the most crucial step in developing cancer vaccines. Different types of therapeutic vaccines have been developed for CRC patients. Cell-based vaccines are either tumor lysate or immune cells modified to present tumor antigens and tumor antigens’ receptors against CRC. Molecular-based vaccines use tumor-specific antigens known as neoantigens to inhibit tumor progression. Vector-based vaccines present tumor antigens using microorganisms in immunogenic or engineered form (191).

Sipuleucel-T (Provenge) is the first autologous immune cell-based vaccine approved by FDA, 2010 (NCT00779402) for prostate cancer. Cell-based vaccines have been investigated for CRC treatment since 1990 (192, 193). Whole cell-based vaccines are the sub-group that comprise the entire tumor cells antigens and would evoke the immune system against tumor-associated antigens. The development of an universal vaccine that could protect any patients is a complex task (194). Cancer stem cells could be isolated from whole tumor cells to develop a cancer stem cell-based vaccine. A recent study demonstrated that the targeted MUC1+ CRC stem cells vaccine significantly increases NK cell infiltration and cytotoxicity. CCSC targeted vaccination promotes the release of INFγ, perforin, and granzyme B, decreasing TGF-β production (195). Furthermore, an allogenic cell base vaccine could be a valuable alternative to an autologous tumor cells vaccine. Allogeneic vaccines based on CRC cell lines (HT-29 and SW-480) were reported to induce anti-tumor immunity in CRC by increasing the NK cells’ cytotoxic activity (186).

In a preclinical study of colorectal liver metastasis, vaccination with CT26-derived tumors treated with mitoxantrone (MTX) improved NK cell and T cell infiltration to the tumor site and improved clinical response (196).

DC-based vaccines were mostly reported in clinical research to be constructed based on CEA antigens. DCs were modified to deliver CEA antigens by two CEA mRNA pulsed DCs, and CEA peptide-loaded DCs approaches. Results demonstrated that the DC vaccination was well tolerated and that NK cells level was increased (197, 198). Moreover, a clinical study of 10 patients suffering from CRC demonstrated that the CEA peptide-loaded DC vaccine could increase CEA- specific CTL and NK cell response (199).

Molecular-based vaccines have also been reported to improve NK cells’ cytotoxicity against colon tumors (200). DNA vaccines are based on direct introduction of a tumoral antigen through plasmids containing the TSA sequence. According to the U.S National library of medicine, 62% of DNA vaccine clinical trials in the United States are assigned to cancer vaccines. A DNA-based vaccine encapsulated in polyplex micelles containing TAA, SART3 (squamous cell carcinoma antigen), and C40L + GMCSF as adjuvant genes stimulated CTLs and NK cells’ efficient immune response in peritoneal metastasis of CT26-derived tumor s (200).

Vector-based vaccines composed of viral-based, bacterial-based, and yeast-based vaccines are high-tech approaches to cancer treatment. Immunogenic viruses will be engineered to express TAAs, and the antigens will be presented to cytotoxic T cells to eliminate cancer progression. It has been reported that systemic activation of NK cells and systemic anti-tumor response occur following viral-based vaccination (201). Recently a randomized phase-II clinical trial showed that CEA-targeted adenoviral vaccine in combination with Avelumab and FOLFOX6 is safe. This treatment generated specific NK cellTCD4^+^/TCD8^+^ and ki67, NKP30^+^. However, the trial failed to show and improvement of PFS (202).

Although therapeutic CRC vaccines have shown considerable capacity in tumor inhibition, more investigation are needed before any clinical use. Recent registered clinical research is based on combination therapy of vaccines with approved immune checkpoint blockades and chemotherapy as standard of care (203). The combination of two different antigen delivery systems in first-line treatment and vaccine booster can inhibit the immune system tolerance against the first vectors when more than one dose of vaccine is needed, especially in high recurrence cancers. The most critical issues in vaccine development are the selection of optimal antigens, adjuvant, and delivery methods. Current registered clinical trials of vaccine therapy in CRC are summarized in **Table 3**.

**Table 3:** The current registered clinical trials of vaccine therapy in colorectal cancer.

Title	Vaccine type	Status	Condition	Characteristics	Trial identifier
Fluorouracil, Semustine, and Vincristine Compared With BCG in Treating Patients	Biological: BCG vaccine	Completed	Colorectal Cancer	Phase 3 -1977	NCT00427570
Cyclophosphamide Plus Vaccine Therapy in Treating Patients With Advanced Cancer	Whole cell based - Allogeneic tumor cell vaccine	Completed	Breast Cancer Colorectal Cancer Kidney Cancer Lung Cancer Malignant Mesothelioma Pancreatic Cancer	Phase 2 -1991	NCT00002475
Vaccine Therapy	Peptide based -Ras peptide cancer vaccine	Completed	Recurrent Colon Cancer Extensive Stage Small Cell Lung Cancer Stage III Pancreatic Cancer Stage III Rectal Cancer	phase 1 -1995	NCT00019006
Vaccine Therapy and Biological Therapy	DC based vaccine -mutant p53 peptide pulsed dendritic cell vaccine	Completed	Breast Cancer Cervical Cancer Colorectal Cancer Lung Cancer Ovarian Cancer Pancreatic Cancer	Phase 2 -1996	NCT00019084
Biological Therapy in Treating Patients With Metastatic Cancer	DC based - CEA RNA-pulsed DC cancer vaccine	Completed	Breast Cancer Colorectal Cancer Extrahepatic Bile Duct Cancer Gallbladder Cancer Gastric Cancer Head and Neck Cancer Liver Cancer Lung Cancer Metastatic Cancer Ovarian Cancer Pancreatic Cancer Testicular Germ Cell Tumor	Phase 1 -1997	NCT00004604
Vaccine Therapy Plus Biological Therapy	Peptide based - Ras peptide cancer vaccine	Completed	Colorectal Cancer Endometrial Cancer Head and Neck Cancer Liver Cancer Lung Cancer Melanoma (Skin) Pancreatic Cancer Testicular Germ Cell Tumor Unspecified Adult Solid Tumor	Phase 2 -1997	NCT00019331
Immunotherapy in Treating Patients with Resected Liver Metastases	Carcinoembryonic antigen RNA pulsed DC cancer vaccine	Completed	Metastatic Colorectal Cancer	Phase 1,2 -1998	NCT00003433
Vaccine Therapy With or Without Interleukin-2	Peptide based -Ras peptide cancer vaccine	Completed	Locally Advanced or Metastatic Colorectal Cancer	Phase 1,2 -1999	NCT00019591
Vaccine Therapy Plus QS21 in Treating Patients	Peptide based -Ras peptide cancer vaccine	Completed	Advanced Pancreatic or Colorectal Cancer	Phase 1 -2000	NCT00006387
Vaccine Therapy in Treating Patients With Metastatic Cancer	Peptide based -MAGE-12 peptide vaccine	Completed	Lung Cancer Adult Soft Tissue Sarcoma Colorectal Cancer Bone Cancer Ovarian Sarcoma Melanoma Colon Cancer Rectal Cancer Breast Cancer Eye Cancer Uterine Sarcoma	Phase 1 -2000	NCT00020267
Vaccine therapy in Treating Patients with Stage II or Stage III Colon	Biological: BCG Vaccine, autologous tumor cell vaccine	Completed	Colorectal Cancer	Phase 1, 2 -2001	NCT00016133
Vaccine Therapy in Treating Patients With Colorectal Cancer Metastatic to the Liver	Anti-body based -monoclonal antibody 11D10 anti-idiotype	Completed	Metastatic Colorectal Cancer	Phase 2 -2001	NCT00033748
Vaccine Therapy	Vector based -viral vaccine -TRICOM-CEA(6D)	Completed	Breast Cancer Colorectal Cancer Gallbladder Cancer Gastric Cancer Head and Neck Cancer Liver Cancer Ovarian Cancer Pancreatic Cancer Testicular Germ Cell Tumor	Phase 1 -2002	NCT00027534
An Open Label Study of a Peptide Vaccine	Peptide based -EP2101	Completed	Colorectal Neoplasms	Phase 1 -2003	NCT00054912
Denileukin Diftitox Followed by Vaccine Therapy	Vector based fowlpox virus -recombinant fowlpox-CEA(6D)/ TRICOM vaccine	Completed	Breast Cancer Colorectal Cancer Lung Cancer Pancreatic Cancer Unspecified Adult Solid Tumor	Phase 1 -2005	NCT00128622
Vaccine Therapy in Treating Patients With Liver or Lung Metastases	DC based vaccine	Completed	Colorectal cancer	Phase 2 -2005	NCT00103142
CEA(6D) VRP Vaccine in patients with advanced or metastatic CEA expressing malignancies	Alpha viral replicon particle *vaccine*	Completed	Colorectal Cancer Breast Cancer Lung Cancer Pancreatic Cancer	Phase 1,2 -2007	NCT00529984
Study of Colon GVAX and Cyclophosphamide	Whole cell based	Completed	Colorectal Cancer Metastatic Cancer	Phase 1 -2008	NCT00656123
Study of the MUC1 Peptide- Poly-ICLC Adjuvant Vaccine	Peptide based	Completed	Advanced colorectal adenoma	Phase 2 -2008	NCT00773097
Immunotherapy With CEA(6D) VRP Vaccine (AVX701)	Alpha viral replicon particle *vaccine*	Completed	Stage III Colon Cancer	Phase 1 -2013	NCT01890213
SGI-110 in Combination With an Allogeneic Colon Cancer Cell Vaccine (GVAX) and Cyclophosphamide (CY)	Whole cell based	Completed	Metastatic Colorectal Cancer	Phase 1 -2014	NCT01966289
Study of GVAX (With CY) and Pembrolizumab	Whole cell based	Completed	MMR-p advanced Colorectal Cancer	Phase 2 -2017	NCT02981524
Pooled Mutant KRAS-Targeted Long Peptide Vaccine Combined With Nivolumab and Ipilimumab	Peptide based	Recruiting	Resected MMR-p Colorectal and Pancreatic Cancer	Phase 1 -2020	NCT04117087
A Study of ELI-002 in Subjects With KRAS Mutated Pancreatic Ductal Adenocarcinoma (PDAC) and Other Solid Tumors	Peptide based -ELI-002 2P	Recruiting	Pancreatic Ductal Adenocarcinoma Colorectal Cancer Non-small Cell Lung Cancer Ovarian Cancer Cholangiocarcinoma Bile Duct Cancer Gallbladder Carcinoma	Phase 1 -2021	NCT04853017
A Vaccine (PolyPEPI1018 Vaccine) and TAS-102 for the Treatment of Metastatic Colorectal Cancer	Peptide based	Recruiting	-Metastatic Microsatellite Stable Colorectal Carcinoma -Stage IV, IVA, IVB, IVC Colorectal Cancer	Phase 1 -2022	NCT05130060
Study of an Individualized Vaccine Targeting Neoantigens in Combination with Immune Checkpoint Blockade	Vector based -Adenoviral,*GRT*- *C901/*GRT-R902	Recruiting	Colorectal Neoplasms	Phase 2 -2022	NCT05456165
Neoantigen-Targeted Vaccine Combined with Anti PD-1 Antibody	Peptide based	Not yet recruiting	Patients With Stage IV MMR-p Colon and Pancreatic Ductal Cancer	Phase 1 -2022	NCT04799431

## NK cells gene modification improves immunogenicity of colorectal cancer

Adoptive manipulated cell therapy has become a highly promising treatment for advanced cancers. Patients affected by Multiple Myeloma and B-lymphoma showed significant results after receiving autologous T cells (204–206). Current T-cell therapy uses gene-modified chimeric antigen receptor (CAR-T). This was approved in August 2017 for the first time by the Food and Drug Administration (207, 208). However, CAR-modified T cells still have several functional and technical limitations. It can be challenging to generate a product for one patient only, and production costs are economically not sustainable for most health care systems. In addition, autologous products require longer time to generate CAR T-cells.

Allogeneic products have the potential to overcome these limitations, and allogeneic HLA-matched T-cells can mediate graft *versus* host disease (GvHD) (209). During the CAR-T cell development NK cell- therapy was also considered. NK cells provide an attractive source of allogeneic cells and have become one of the hopes of the CAR engineering approach. Allogeneic NK cells with a short life span do not cause GvHD and also have less long-term adverse events (210–212). Furthermore, donor selection is based on killer cell immunoglobulin receptor (KIR)-ligand mismatch with the recipient or haplotype B KIR gene, which could be beneficial in allogeneic stem cell transplantation (213). CAR NK cell generation has been based on the CAR-T cell platform (comprising CD3ζ and T cell co-stimulatory molecules). It has been shown that these cells target cancer cells with the desired specificity and effectiveness (214). Therefore, CAR-NK-based therapy has been performed on CRC, showing that EpCAM-CAR-NK-92 cells combined with Regorafenib suppress EpCAM-positive tumor xenografts (215). Furthermore, Masayuki Shiozawa et al. demonstrated anti-CEA-CAR NK-92MI cells in a CEA-dependent manner recognized and lysed high CEA-expressing tumor cells (215, 216) and NKG2D CAR mRNA-engineered NK cells significantly improved the cytolytic activity of NK cells against tumors (217). Preclinical models are crucial stages that recapitulate the individual tumor phenotype. The organoid culture system allows long-term *ex vivo* expansion of gastrointestinal stem cells in a 3D extracellular matrix. In a study by Theresa E Schnalzger et al., CRC organoid was admitted to evaluate the performance of EpCAM-CAR-NK-92 and FRIZZLED-CAR-NK-92. This 3D platform is useful for evaluating CAR-engineered lymphocytes (218). Furthermore, the recent development and efficient CRISPR/Cas9 genome-editing technologies have accumulated NK cell properties and offered new opportunities to increase their susceptibility to NK surveillance. Lanlan Gao et al. demonstrated up regulation of CXCR2 and IL-2 *Via* CRISPR-Cas9 improved NK-92 cell anti-tumor effects, and survival time was significantly prolonged as cellular immunotherapy for CRC (219).

Engineered NK cells are the next generation of immune cell therapy products with enhanced proliferation and homing capacity and blocked suppressing signals that will enhance their tumor killing properties.

## NK cell-based clinical trial of colorectal cancer

NK cell-based therapies are well developed for hematological malignancies such as Acute Myeloid Leukemia (AML) (220, 221). The FDA and EMA have represented ONKord as an off-shelf orphan drug for AML patients: the allogeneic partial HLA-matched NK cells derived from UCB-CD34 ^+^ progenitors.

Despite the successes in hematologic malignancies, NK-based therapies for CRC as a solid tumor are associated with challenges. The most difficult are NK cell source and *ex vivo* expansion, lymphocyte infiltration, and tumor escape from the immune surveillance. These challenges have led to fewer trials on this cancer, which has been rising in recent years (**Table 4**). iPSC- differentiated NK cells (iPSC-NK) are homogeneously differentiated and more suitable than NK-92. Other clinical trials (NCT03841110 and NCT04106167) examined the safety and efficacy of these cells under the name FT500 against CRC.

**Table 4:** Ongoing clinical trials of therapeutic NK cells in CRC.

Agent	Cell source	Treatment approach	Malignancy	Year, Study phase (status)	Trial identifier
Adoptive NK cell					
NK	Cord blood	In combination with Cetuximab	CRC	2021, Phase 1 (Not yet recruiting)	NCT05040568
HSP-70 activated NK cells	PBMC	Autologous NK cell	Colon and lung cancer	2004, Phase 1Complete	(222)
NK	PBMC	Evaluation of safety following allogenic hematopoietic stem cell stem cell transplantation	CRC, HCC, RCC, B-CLL	2009, Phase 1, Complete	(223)
NK	PBMC	In combination with conventional treatment	CRC	2021, Phase 1, Complete	(224)
NK	PBMC	Autologous NK cell	Advanced digestive cancer	2015, Phase 1, Complete	(225)
NK	PBMC	In combination with IgG1 antibody	Gastric and Colorectal cancer	2018, Phase 1, Complete	(226)
NK	PBMC	Combined with Cetuximab	Gastrointestinal carcinoma	2018, Phase 1, Complete	(227)
FATE-NK100	PB NK cells	Monotherapy and in combination with trastuzumab	Advanced Solid Tumors	2017, Phase 1 (not recruiting)	NCT03319459
FT500	iPS	Monotherapy and in Combination with Immune Checkpoint Inhibitors	Advanced Solid Tumors	2019, Phase 1 (Recruiting)	NCT03841110
FT500	iPS	Evaluation of long-term safety and efficacy	Solid tumors	2019, (recruiting)	NCT04106167
CB-NK cells	–	Evaluation of safety and activity of combination with cetuximab	Colon Cancer	2021, phase 1b, Recruiting	NCT05040568
FT536	–	Dose-finding study in Combination With Monoclonal Antibodies	Advanced Solid Tumors	2022, phase 1, Recruiting	NCT05395052
DKC	–	Evaluate the safety of autologous dendritic killer cell (DKC)	Solid Tumors	2016, Phase 1 (not recruiting)	NCT02882659
NKT	PBMC	Evaluation of Clinical Efficacy and Safety	Advanced Solid Tumor	2015, Phase 1/2 (Recruiting)	NCT02562963
DC-CIK	–	Evaluate the efficacy	Colorectal Cancer	2013, Phase II, Unknown	NCT01839539
NK	–	Evaluate Safety in Combination With Interleukin-2 (IL-2) and Transforming Growth Factor Beta (TGFbeta) Receptor I Inhibitor Vactosertib	Colorectal Cancer	2022, phase 1 Not yet recruiting	NCT05400122
NKG2D CAR - NK	PBMC	Evaluate the safety and efficacy	Metastatic Colorectal Cancer	2019, Open label pilot study	(217)
CAR-pNK	–	Anti-MUC1 CAR-pNK cells	Refractory Solid Tumor	2016, Phase 1/2 (Recruiting)	NCT02839954
CAR-NKG2D	–	Anti-NKG2D CAR-pNK cells	Refractory Metastatic Colorectal Cancer	2022, phase 1 (Recruiting)	NCT05213195
SNK01	Autologous non-genetically modified	Safety and Efficacy of SNK01 in Combination with Trastuzumab or Cetuximab	Advanced HER2 or EGFR	2020, Phase1/2 (Trial not initiated)	NCT04464967
ACE1702	–	Evaluate the safety and tolerability, pharmacokinetics, pharmacodynamics, and preliminary efficacy	HER2-expressing Solid Tumors	2020, Phase 1 (Recruiting)	NCT04319757
NK cell augment					
Lidocaine	–	Intravenous lidocaine in CRC resection will preserve NKs activity	CRC	2013, Phase 4 (Recruiting)	NCT01841294
Aerobic Exercise	–	Exercise increases the activity of NK cells	CRC Stage IV	2021, Not Applicable (Recruiting)	NCT04715061

The first human study of NK cell transfer in CRC showed stable disease in metastasis and progression stages and was also reported to be safe with no side effects, mainly including GvHD (222–224). Furthermore, a phase-I clinical trial revealed the safety of autologous NK cell therapy and was reported tolerable in patients suffering from CRC who had failed previous standard therapy. Autologous NK cells were administered dose-escalating (dose 0.5 × 109, 1.0 × 109, 2.0 × 109 cells/injection) three times/week. The results demonstrated that adoptive NK cell monotherapy caused no clinical responses besides safety and was undesirable for patients. To improve their efficacy, combining approaches with other immune therapy agents should be considered (225).

It has become increasingly clear that NK therapy alone has limited efficacy in solid tumors. Most studies have shifted to engineered NKs and combined therapies.

Takeshi Ishikawa et al. performed a human study (N = 9) of expanded NK cells in combination with IgG1 antibody (Trastuzumab, Cetuximab). Patients received NK cells after three days of IgG1 antibody administration were infused with expanded NK cells in three steps, at doses of 0.5 × 10^9^, 1.0 × 10^9^, and 2.0 × 10^9^ cells/injection at tri-weekly intervals. A decrease in tumor size in three patients and raised whole blood IFNγ production after combination therapy was observed (226).

Allogeneic NK cells combined with Cetuximab were administered in metastatic colorectal carcinoma (N = 6) to evaluate the safety and efficacy of NK cell delivery in the phase-I clinical trial. NK cells were administrated, followed by high-dose IL-2 (3×10^6^, 8×10^6^, and 12×10^6^ NK cells/kg). Reportedly, NK cell-Cetuximab combination approach was well tolerated. However, clinical responses should be further investigated (227). Chimeric antigen receptor (CAR) -carrying cells have been shown to be effective in hematologic malignancy.

In comparison to CAR based T cell therapy, allogeneic CAR-NK cell therapy caused no GvHD (228, 229), accompanied by a shorter final production time (230, 231), and also the final product would be “off-the-shelf” (232). LinXiao et al. performed an open-label pilot study (NCT03415100) to assess the safety of NKG2D CAR mRNA-engineered NK Cells to improve their cytolytic activity against metastatic CRC patients. No serious adverse effects (≥grade 3 adverse events) existed in any of the three patients except grade 1 cytokine release syndrome. CAR-NK cells augmented NK-based therapy when they were administered intra -peritoneal in a dose-escalation manner to reduce off-target risks (217). Moreover, a clinical trial was recently registered (NCT02839954) to administer anti-MUC1 CAR-NK cells in patients suffering from solid tumors, including colorectal cancer. Additionally, a phase-I clinical study (NCT04319757) is recruiting patients to investigate the safety and preliminary efficacy of anti-HER2 oNK cells (ACE1702) against human HER2-expressing solid tumors as an off-the-shelf NK cell product.

The infiltration and active persistence of NK cells and genetic manipulation have been among researchers’ priorities. In recent years, the identification of checkpoint inhibitors has led to immunotherapy development, which is being evaluated in combination (NCT03841110) with FT500 to inhibit cancer immunity. Ongoing clinical trials of therapeutic NK cells in CRC have been reviewed in **Table 4**.

## Conclusion

NK cell therapy has been widely adopted as an efficient cancer treatment, however; the limitation and challenges, especially in solid tumors, are indisputable. The major limitations reported are the non-targeted responses of NK cells, the immune suppressive tumor microenvironment, and the NK cell infiltration barriers to the tumor site. Genetic engineering strategy has developed chimeric antigen receptors NK cells; in addition, immune checkpoints recognition and development of immune blockade molecules effectively improved the adaptive NK cell therapy effectiveness. The use of NK cells to target cancer cells and understanding the NK cell–cancer interactions is constantly evolving.

## Author contributions

The authors confirm responsibility for the following: study conception and design, data collection, analysis and interpretation of results, and manuscript preparation. All authors contributed to the article and approved the submitted version.
